# Deep Spectrogram Learning for Gunshot Classification: A Comparative Study of CNN Architectures and Time-Frequency Representations

**DOI:** 10.3390/jimaging11080281

**Published:** 2025-08-21

**Authors:** Pafan Doungpaisan, Peerapol Khunarsa

**Affiliations:** 1 Faculty of Industrial Technology and Management, King Mongkut’s University of Technology North Bangkok, Bangkok 10800, Thailand; pafan.d@itm.kmutnb.ac.th; 2 Faculty of Science and Technology, Uttaradit Rajabhat University, Uttaradit 53000, Thailand

**Keywords:** gunshot detection, RGB spectrogram, convolutional neural networks (CNNs), image processing, time-frequency analysis, deep learning, audio classification, computer vision

## Abstract

Gunshot sound classification plays a crucial role in public safety, forensic investigations, and intelligent surveillance systems. This study evaluates the performance of deep learning models in classifying firearm sounds by analyzing twelve time–frequency spectrogram representations, including Mel, Bark, MFCC, CQT, Cochleagram, STFT, FFT, Reassigned, Chroma, Spectral Contrast, and Wavelet. The dataset consists of 2148 gunshot recordings from four firearm types, collected in a semi-controlled outdoor environment under multi-orientation conditions. To leverage advanced computer vision techniques, all spectrograms were converted into RGB images using perceptually informed colormaps. This enabled the application of image processing approaches and fine-tuning of pre-trained Convolutional Neural Networks (CNNs) originally developed for natural image classification. Six CNN architectures—ResNet18, ResNet50, ResNet101, GoogLeNet, Inception-v3, and InceptionResNetV2—were trained on these spectrogram images. Experimental results indicate that CQT, Cochleagram, and Mel spectrograms consistently achieved high classification accuracy, exceeding 94% when paired with deep CNNs such as ResNet101 and InceptionResNetV2. These findings demonstrate that transforming time–frequency features into RGB images not only facilitates the use of image-based processing but also allows deep models to capture rich spectral–temporal patterns, providing a robust framework for accurate firearm sound classification.

## 1. Introduction

Global firearm-related violence has escalated in recent years, amplifying the demand for rapid, AI-based gunshot detection systems. These technologies analyze acoustic signatures—frequency, amplitude, and temporal waveforms—to differentiate various firearm discharges in real time. AI-enabled systems offer law enforcement a non-invasive, automated alternative to traditional forensic ballistics, delivering location-based alerts for immediate response.

In 2022, the United States experienced over 650 mass shootings—defined as incidents involving four or more victims, excluding the shooter—with total firearm-related deaths surpassing 43,000. This alarming trend has driven urban centers to adopt advanced acoustic surveillance. ShotSpotter, deployed in more than 100 U.S. cities (including Chicago, New York, and San Francisco), utilizes sensor networks to triangulate gunfire and dispatch alerts in under a minute. Independent studies report up to 97% system accuracy in controlled conditions, though real-world performance varies. Estimated annual deployment and maintenance costs can exceed $65,000 per square mile [1].

Research on ShotSpotter reveals mixed outcomes. Some evaluations note faster police response and improved evidence collection—including assistance in incidents not reported via 911—but criminological analyses suggest no statistically significant reduction in violent crime or firearm homicides. Critics also raise concerns over false positives, racial profiling, and high infrastructure costs, leading some cities to terminate contracts amid concerns over their efficacy [2].

Internationally, regions in Latin America face severe gun violence. Brazil, for example, recorded nearly 38,800 homicides in 2024—an intentional homicide rate of 17.9 per 100,000—with firearms implicated in the majority of cases [3].

Despite being the country’s lowest rate in over a decade, gun violence remains widespread, driven by organized crime, firearm accessibility, and social inequality [4].

Therefore, gunfire threat detection systems are critically important—especially in high-risk urban environments where rapid law enforcement response can significantly reduce the severity of incidents and enhance public safety.

In recent years, artificial intelligence (AI), machine learning (ML), and deep learning (DL) have made substantial progress in the field of audio signal classification, particularly in gunshot detection. A core principle of this research involves applying image processing and pattern recognition techniques to audio data. This is accomplished by converting audio signals into time-frequency representations, which can be visualized as images. These spectrogram-like visualizations enable the use of deep-learning models—originally developed for computer vision tasks—to effectively analyze and classify firearm sounds [3,5,6].

Time-frequency features provide a comprehensive representation that integrates both the time and frequency domains, unlike traditional methods that analyze these aspects in isolation. Modern techniques such as spectrograms, wavelet transforms, and Mel-Frequency Cepstral Coefficients (MFCCs) enable models to extract deep temporal and spectral characteristics. Spectrograms, in particular, as visual representations of a signal’s frequency distribution over time, have become an exceptionally suitable tool for convolutional neural network (CNN)-based pattern recognition in gunshot classification [7].

To further strengthen the link with image processing and create a more complex, multi-dimensional input, the concept of the RGB Spectrogram is applied. In this approach, each color channel (Red, Green, Blue) of an image is used to represent a different time-frequency feature. For instance, the Red (R) channel might represent the standard Mel spectrogram, the Green (G) channel could represent the delta features (the rate of spectral change), and the Blue (B) channel could represent the delta-delta features (the rate of acceleration of that change). This process effectively creates a “color image” from the audio signal, providing richer information than a standard grayscale spectrogram. It allows a CNN to learn more intricate patterns, thereby improving its ability to effectively distinguish gunshots from environmental noise [5,6,8,9].

The efficacy of CNN models in processing image-like data has been extensively proven in other fields, most notably in medical imaging for the analysis of MRI and CT scans [10] and pathology detection [11]. This success validates the application of the same principles to the analysis of audio spectrograms. Deep-learning models can automatically extract spatial and temporal features from these spectrograms, surpassing traditional ML algorithms that often depend on manual feature engineering [12].

Therefore, this research focuses on the exploration and application of AI and DL techniques, centered on the transformation of gunshot audio signals into image-based spectrograms (including the RGB spectrogram concept) for use as input to image-classification models. This methodology aims not only to enhance classification accuracy but also to improve the system’s robustness against confounding noises with similar acoustic characteristics, such as fireworks or vehicle backfires, addressing a critical challenge for the reliable, semi-controlled outdoor environment deployment of automated gunshot detection systems.

This study presents a comprehensive evaluation of deep-learning-based firearm sound classification using time-frequency representations. By converting 12 spectrogram types—including perceptual, Fourier-based, feature-specific, and wavelet-based methods—into RGB images and feeding them into six pretrained CNN architectures, the study establishes a reproducible benchmark and demonstrates the feasibility of applying deep image models to acoustic recognition tasks. The investigation lays a foundation for future work in intelligent acoustic surveillance systems.

Key Contributions.

The key contributions of this study are summarized as follows:

- A systematic benchmark of 12 time-frequency spectrogram representations—transformed into RGB images—for gunshot classification.
- Comparative evaluation of six state-of-the-art pretrained CNN architectures (ResNet18, ResNet50, ResNet101, GoogLeNet, Inception-v3, and InceptionResNetV2) under consistent 5-fold cross-validation, which serves not only as a performance evaluation method but also as a regularization strategy to reduce overfitting in training with a relatively small dataset.
- Identification of optimal spectrogram–CNN pairs (e.g., CQT + InceptionResNetV2) achieving up to 95.81% accuracy, 0.9447 F1-score, and 0.9957 AUC.
- Inclusion of misclassification analysis and computational cost comparison to support real-time or edge deployment.

The remainder of this paper is organized as follows: Section 2 formulates the problem and defines the operational constraints of firearm sound recognition. Section 3 describes the dataset, spectrogram generation methods, CNN architectures, and experimental settings. Section 4 presents the classification outcomes, misclassification analysis, and computational cost evaluation. Finally, Section 5 summarizes the findings and outlines potential directions for future work.

## 2. Problem Formulation and Constraints

This study aims to accurately classify various types of firearms by analyzing the acoustic signatures of their gunshots captured in audio recordings. To advance the field of gunshot sound classification, the research proposes the development of a high-performance classification system that integrates advanced audio feature extraction techniques with state-of-the-art convolutional neural network (CNN) architectures. The system will utilize a range of spectrogram representations—including the Cochleagram, which models the human auditory response, as well as Constant-Q Transform (CQT), Fast Fourier Transform (FFT), Mel Spectrogram, Mel-Frequency Cepstral Coefficients (MFCC), Reassigned Spectrogram, Spectral Contrast, Short-Time Fourier Transform (STFT), and Wavelet Spectrograms. By combining these diverse spectrogram features with deep-learning models, the proposed approach is expected to improve classification accuracy and robustness across different firearm types.

Accurately classifying firearm types based on gunshot audio presents significant challenges due to the subtle acoustic differences between firearms. These differences are shaped by variables such as firearm design, ammunition type, and environmental recording conditions. Traditional classification methods often fail to capture the nuanced spectral and temporal features required for reliable identification. To address this limitation, the proposed study leverages advanced audio feature extraction using Mel spectrograms in conjunction with multiple convolutional neural network (CNN) architectures. This approach is designed to enhance classification accuracy and robustness by effectively learning discriminative patterns in gunshot audio signals.

A central challenge in gunshot classification is distinguishing between firearms with acoustically similar signatures, a task further complicated by external influences such as recording environment, distance, and background noise. These subtle variations often elude traditional classification techniques, resulting in reduced accuracy and inconsistent performance. This research addresses these limitations by employing advanced methods capable of capturing and interpreting fine-grained acoustic features. Through the integration of sophisticated audio representations and deep-learning models, the proposed system aims to deliver a more accurate and reliable solution for firearm classification based on gunshot sounds.

### 2.1. Research Objectives

- **Enhance Firearm Sound Classification Accuracy:** To develop an advanced system that significantly improves the accuracy of firearm identification from gunshot sounds by leveraging time-frequency spectrogram representations and state-of-the-art convolutional neural network (CNN) architectures.
- **Evaluate the Discriminative Power of Spectrogram Types:** To systematically assess and compare 12 distinct spectrogram techniques—including perceptual, Fourier-based, feature-specific, and wavelet-based representations—to determine their effectiveness in capturing relevant acoustic features for gunshot classification.
- **Assess and Benchmark CNN Architectures:** To train and evaluate multiple CNN models—such as ResNet18/50/101, Inception-v3, InceptionResNetV2, and GoogLeNet—on RGB spectrogram inputs, identifying which architectures deliver optimal performance in terms of accuracy, generalization, and computational efficiency.

### 2.2. Scope of Research

This research focuses on the development and evaluation of a firearm sound classification system using deep learning and advanced spectrogram-based audio analysis. The scope of this study is defined by the following components:

- **Dataset and Firearm Types:** The study utilizes a dataset consisting of 2148 gunshot audio recordings collected in semi-controlled, real-world conditions. The dataset includes four distinct firearm types: Smith & Wesson (.38), Glock 17 Gen3 (9 mm), Remington 870 (12-gauge), and Ruger AR-556 (.223 caliber) [13].
- **Spectrogram-Based Feature Extraction:** Twelve time-frequency spectrogram types are examined, including perceptual-based (e.g., Mel, Bark, Cochleagram, CQT), Fourier-based (e.g., FFT, STFT, Reassigned), feature-specific (e.g., MFCC, Chroma, Spectral Contrast), and wavelet-based representations. These are converted into RGB images to facilitate their use with image-based deep-learning models.
- **Deep-Learning Models:** The study evaluates six CNN architectures—ResNet18, ResNet50, ResNet101, GoogLeNet, Inception-v3, and InceptionResNetV2—using transfer learning. Each model is trained and fine-tuned to classify the spectrogram images into the corresponding firearm categories.
- **Performance Evaluation:** Model performance is evaluated using metrics such as accuracy, precision, recall, F1-score, and area under the curve (AUC). A 5-fold cross-validation technique is employed to ensure the robustness and generalizability of the results.

This study does not cover aspects such as real-time detection, sequential gunshot analysis, or integration with multimedia surveillance systems. The scope is limited to static classification of individual gunshot audio events using deep spectrogram learning techniques.

Inspired by work on transient noise removal using EMD and wavelet-based filtering [14], future work could explore the integration of denoising techniques before spectrogram generation to enhance signal clarity for CNNs. Moreover, the approach of learning disease patterns from leaf images [15,16] demonstrates the potential of CNNs in interpreting domain-specific visual representations. Similarly, our study transforms time-frequency features into RGB spectrograms, enabling CNNs to extract discriminative patterns akin to leaf texture or disease markings.

### 2.3. Technical Constraints

- **Software and Tools:** This research employed MATLAB R2024a, utilizing its Deep-Learning Toolbox and Audio Toolbox for developing and training CNN-based models. The toolboxes offer advanced functions for audio processing, including Mel spectrogram computation, and feature extraction, as well as GPU acceleration to optimize training. MATLAB’s integrated environment provides an efficient platform for audio classification tasks, particularly gunshot sound detection.

## 3. Materials and Methods

This research focuses on utilizing artificial intelligence (AI), deep learning, and image-classification techniques to recognize gunshot sounds through spectrogram analysis. Spectrograms visually represent an audio signal’s frequency spectrum over time, enabling deep-learning models to extract meaningful patterns. The core approach is to treat spectrograms as image-based features and apply convolutional neural networks (CNNs)—a technique widely used in computer vision—for firearm sound classification. Each spectrogram type offers unique insights into the signal’s characteristics by capturing critical time-frequency features that enhance classification accuracy.

To match the standard input format of image-based CNNs, each spectrogram is converted into a three-channel RGB image using perceptually informed colormaps (e.g., Jet, Viridis). This transformation enhances visual resolution and enables transfer learning from CNN models pretrained on RGB image datasets such as ImageNet. In this study, we focus exclusively on the three-channel RGB representation, which provides rich spatial and spectral features for deep-learning models. Alternative formats, such as grayscale spectrograms or other multi-channel mappings, were not explored in this work and are left for future investigation.

As shown in Table 1, spectrograms are particularly effective for analyzing non-stationary signals, as they reveal how frequency components evolve. Different spectrogram types emphasize distinct acoustic features, making them valuable for a range of applications, including speech recognition, music analysis, and environmental sound classification. For example, the Bark spectrogram is aligned with human auditory perception, while the Chroma Spectrogram emphasizes tonal structures. The Cochleagram simulates the human ear’s response, while others—such as Constant-Q Transform (CQT), Fast Fourier Transform (FFT), Mel, Mel-Frequency Cepstral Coefficients (MFCC), Reassigned, Spectral Contrast, Short-Time Fourier Transform (STFT), and Wavelet—highlight specific frequency and temporal dynamics.

A major challenge in this research is selecting the most suitable spectrogram type for firearm classification. Each method emphasizes different spectral properties, which impact how effectively CNN models can learn and distinguish gunshot sounds. Thus, the task involves optimizing feature selection while ensuring computational efficiency.

This study employs CNNs to process spectrograms as two-dimensional images, enabling the automatic extraction of complex temporal and spectral patterns. Compared to traditional audio analysis methods, CNN-based classification provides superior accuracy and robustness, particularly in challenging acoustic environments.

### 3.1. Data and Tools

As shown in Table 2, the firearm spectrogram dataset includes models such as the Smith & Wesson (.38), Glock 17 Gen3 (9 mm), Remington 870 (12-gauge), and Ruger AR-556 (.223 caliber). Each firearm exhibits distinct acoustic signatures. By applying CNNs to their spectrogram representations, this research aims to improve firearm identification accuracy, contributing to advancements in forensic analysis and security surveillance systems.

- **Firearm Types:** The research focuses on four specific firearm types: Smith & Wesson (.38), Glock 17 Gen3 (9 mm), Remington 870 (12-gauge), and Ruger AR-556 (.223 caliber).
- **Dataset:** The dataset provided by Kabealo et al. [13] is a comprehensive audio collection featuring gunshots from multiple firearms and orientations. The data were gathered in a semi-controlled real-world environment using edge devices strategically placed in and around an outdoor firing range. This approach ensured the capture of diverse acoustic signatures by recording gunshots from various angles.
  The recordings were made in stereo at a sampling rate of 44.2 kHz with a bit rate of 128 kbps, resulting in a dataset of 2148 audio files, each precisely timestamped to mark gunshot occurrences. This dataset serves as a valuable resource for studying gunshot sounds in a variety of conditions.
- **Feature and Model Selection:** Concentrate on the selected audio features and CNN architectures, while recognizing that other features or models may exist but are not included in this study.

### 3.2. Methodology

**Figure 1:**
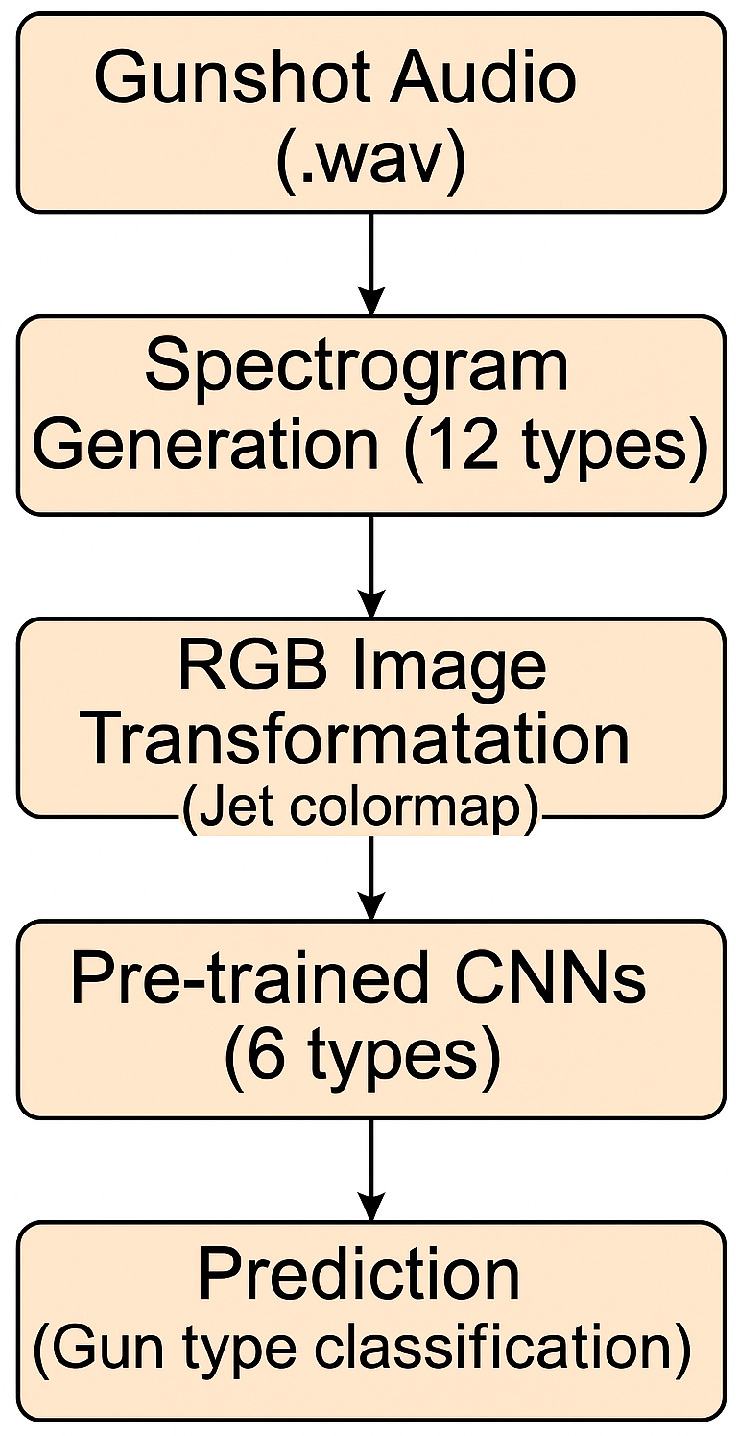
Proposed pipeline for firearm sound classification using deep spectrogram learning.

- **Enhance Classification Accuracy:** Develop a system that improves the precision of firearm identification from gunshot sounds by utilizing advanced audio features and modern CNN architectures. The focus is on achieving high accuracy through the detailed analysis of the unique acoustic properties of different gunshots.
- **Evaluate Audio Features:**
  This study investigates firearm sound identification through deep learning and spectrogram-based image classification. Gunshot audio recordings are converted into spectrograms, enabling a visual representation of acoustic data. Each spectrogram type emphasizes distinct frequency and temporal features, contributing to classification performance.
  As illustrated in Figure 1, the proposed pipeline begins with raw gunshot audio, followed by the generation of 12 spectrogram types. These are transformed into RGB images using the Jet colormap and subsequently classified using six pretrained CNN architectures. The final output is the predicted gun type.
  The 12 spectrogram types used in this study employ diverse computational methods, filter banks, and frequency representations. Their applicability spans various domains, including speech recognition, music genre classification, and forensic audio analysis.
  – **Perceptual-Based Spectrograms**: The **Bark, Mel, Cochleagram, and CQT spectrograms** are inspired by human auditory perception. The **Bark spectrogram** applies a Bark-scaled gammatone filterbank with 64 filters, simulating the frequency response of the human ear. The **Mel Spectrogram**, with 64 Mel bands, represents frequencies in a way that aligns with human pitch perception. The **Cochleagram Spectrogram** employs Gammatone-based ERB filters, capturing the auditory response more precisely. The **CQT Spectrogram (Constant-Q Transform)** uses 24 bins per octave, making it particularly effective for pitch-based analysis in music.
  – **Fourier-Based Spectrograms**: **STFT, FFT, and Reassigned Spectrograms** rely on Short-Time Fourier Transform (STFT) principles with a 512-sample window size and Hamming window function. The **FFT Spectrogram** extracts frequency components using the Fast Fourier Transform (FFT). The **STFT Spectrogram** presents a detailed time-frequency representation with logarithmic scaling for enhanced amplitude contrast. The **Reassigned Spectrogram** improves time-frequency resolution by relocating spectral energy, making it ideal for transient sound analysis.
  – **Feature-Specific Spectrograms**: **MFCC Spectrogram** extracts Mel-Frequency Cepstral Coefficients (MFCCs), a widely used feature set in speech and audio classification. The **Spectral Contrast Spectrogram** calculates contrast between spectral peaks and valleys across six frequency bands, highlighting harmonic structures. The **Chroma Spectrogram** maps energy across 12 Chroma bins, making it effective for chord detection and musical key estimation.
  – **Wavelet-Based Spectrogram**: The **Wavelet Transform Spectrogram** employs Morlet wavelets for adaptive time-frequency resolution, offering better analysis for non-stationary signals.
  Most spectrograms use **Jet colormap** and **logarithmic scaling**, ensuring clarity in visual representation. These diverse spectrograms provide powerful tools for different applications, from music genre classification to gunshot identification and environmental sound analysis [17,18,19,20,21,22,23,24,25,26].
- **Assess CNN Architectures:** This study aims to identify the most effective deep-learning model for firearm sound classification by training and evaluating multiple convolutional neural network (CNN) architectures on spectrogram images of gunshot sounds. Each model is assessed based on its classification accuracy across different firearm types, leveraging distinct architectural features that enhance learning and performance.
  To achieve this, we experimented with various CNN architectures, including ResNet50, ResNet101, ResNet18, Inception-v3, GoogLeNet, and Inception-ResNet-v2. These models were selected for their proven ability to capture complex patterns in image and audio data, with each architecture offering unique advantages in terms of network depth, parameter efficiency, and feature extraction capabilities. The experiments were conducted using MATLAB, utilizing its built-in support for pretrained deep-learning networks to streamline model implementation and optimization [27,28,29,30,31,32,33,34,35].
  – **Pretrained GoogLeNet**: Pretrained GoogLeNet refers to a deep convolutional neural network architecture that has already been trained on the large-scale ImageNet dataset, which contains over 1.2 million labeled images spanning 1000 object categories. Rather than training from scratch, a pretrained GoogLeNet model allows researchers to reuse learned features for new image-classification tasks through transfer learning. This is particularly useful in domains with limited labeled data, such as medical imaging, spectrogram analysis, and environmental sound classification.
    GoogLeNet was introduced by Christian Szegedy and his team at Google Research in 2014 in the paper “Going Deeper with Convolutions” [36], where it won the ImageNet Large-Scale Visual Recognition Challenge (ILSVRC) with a top-5 error rate of just 6.67%. Its core innovation lies in the Inception module, which applies 1 × 1, 3 × 3, and 5 × 5 convolutions, as well as max-pooling, in parallel to efficiently capture multi-scale features. Despite its 22-layer depth, GoogLeNet significantly reduces parameters compared to traditional networks.
    The main benefits of pretrained GoogLeNet include faster training, better generalization on small datasets, and reduced resource demands. However, its performance may degrade when applied to domains far removed from natural images, and newer architectures like ResNet or Vision Transformers have since surpassed it in flexibility and accuracy [37,38,39,40].
    This research employs a pretrained GoogLeNet model, originally trained on the ImageNet dataset, to classify spectrogram images of gunshot audio using transfer learning. GoogLeNet is a 22-layer deep convolutional neural network that integrates the Inception module, which allows multiple convolution operations in parallel to extract multi-scale features efficiently. It is designed to accept RGB images with an input size of 224 × 224 × 3, making it highly suitable for image-based tasks such as time-frequency spectrogram analysis.
    In this study, the GoogLeNet model is loaded using the MATLAB function *googlenet*, which retrieves the pretrained architecture. The original output layers—loss3-classifier, prob, and output—are removed and replaced with a new fully connected layer, a SoftMax layer, and a classification layer. The newly added fully connected layer uses higher learning rate factors (WeightLearnRateFactor and BiasLearnRateFactor set to 20) to accelerate adaptation to the new task. These layers are connected to the pool5-drop_7x7_s1 layer of the original network. Input images are resized using the augmentedImageDatastore function to match the expected input dimensions. The network is trained using the Adam optimizer with a mini-batch size of 32, up to 100 epochs, and an initial learning rate of 0.0001. The model thus retains learned features from ImageNet while adapting to classify gunshot-related spectrogram images efficiently.
  – **Pretrained InceptionResNetV2**: Pretrained InceptionResNetV2 is a deep convolutional neural network that combines two powerful architectures—Inception and ResNet—and is trained on large-scale datasets such as ImageNet. This model is designed to extract rich and multi-scale features efficiently through the Inception module while leveraging residual connections to improve gradient flow and facilitate the training of very deep networks. As a pretrained model, it can be directly applied or fine-tuned for new tasks like image classification, object detection, or spectrogram-based audio analysis using transfer learning. This significantly reduces training time and computational costs while enhancing performance, especially when training data are limited.
    The architecture was introduced by Christian Szegedy and his team at Google Research in the 2016 paper titled “Inception-v4, Inception-ResNet and the Impact of Residual Connections on Learning” [41]. It was designed to combine the parallel convolution strategy of Inception modules with the identity mapping used in ResNet for more stable and faster convergence.
    The main advantages of InceptionResNetV2 include high accuracy, efficient feature extraction, and strong generalization across different domains. However, the model is computationally heavy and may not be suitable for real-time or low-resource applications. Despite this, it remains a top-performing architecture widely used in research and industry [42,43,44].
    In this study, the pretrained **InceptionResNetV2** model is employed for the classification of gunshot audio spectrogram images using transfer learning. This deep convolutional neural network, originally trained on the ImageNet dataset, combines the strengths of Inception modules for multi-scale feature extraction and residual connections for improved gradient propagation. The network accepts RGB images of size 299×299 as input. To adapt the model to the target classification task, the final layers—predictions, predictions_softmax, and ClassificationLayer_predictions—are removed and replaced with a new fully connected layer, a softmax layer, and a classification layer. The newly added fully connected layer is configured with the number of output classes and is assigned increased learning rate factors (WeightLearnRateFactor and BiasLearnRateFactor set to 20) to accelerate training on new data. The modified layers are connected to the avg_pool layer in the original architecture. Input images are resized automatically using augmentedImageDatastore to match the required input dimensions. The model is trained using the Adam optimizer with a mini-batch size of 32, an initial learning rate of 0.0001, and a maximum of 500 training epochs. This approach allows the model to retain its powerful pre-learned visual features while adapting efficiently to the domain-specific spectrogram classification task.
  – **Pretrained Inception-v3**: In this study, the pretrained **Inception-v3** model is applied for the classification of gunshot audio spectrogram images using transfer learning. Inception-v3 is a deep convolutional neural network architecture trained on the ImageNet dataset and designed to accept RGB input images with a resolution of 299×299 pixels. To adapt the model to the target dataset, the original output layers—predictions, predictions_softmax, and ClassificationLayer_predictions—are removed. These are replaced with a new fully connected layer, a softmax layer, and a classification layer, where the number of output units in the fully connected layer corresponds to the number of spectrogram classes. To accelerate training of these new layers, the WeightLearnRateFactor and BiasLearnRateFactor are both set to 20. The new layers are connected to the avg_pool layer of the original network. Input spectrogram images are automatically resized using the augmentedImageDatastore function. The training is configured with the Adam optimizer, using a mini-batch size of 32, an initial learning rate of 0.0001, and a maximum of 500 training epochs. This transfer learning approach enables efficient reuse of learned features from Inception-v3 while adapting the model to classify spectrogram images representing gunshot sounds [45,46,47].
  – **Pretrained ResNet-18, ResNet-50, and ResNet-101**: In this study, the pretrained **ResNet18** model is employed for classifying gunshot audio spectrogram images using transfer learning. ResNet18, ResNet-50, and ResNet-101 comprise a convolutional neural network composed of 18 layers with skip (residual) connections that facilitate the training of deeper networks by mitigating the vanishing gradient problem. The model, originally trained on the ImageNet dataset, accepts input images with a resolution of 224×224×3 (RGB). To adapt the network to the target classification task, the final three layers—fc1000, prob, and ClassificationLayer_predictions—are removed and replaced with a new fully connected layer, a softmax layer, and a classification output layer. The new fully connected layer is set to match the number of classes in the dataset and is configured with WeightLearnRateFactor and BiasLearnRateFactor values of 20 to speed up learning in the new layers. The network is trained using the Adam optimizer with a mini-batch size of 32, an initial learning rate of 0.0001, and a maximum of 500 training epochs. Input images are resized automatically using augmentedImageDatastore. This configuration allows the model to retain useful features learned from ImageNet while adapting efficiently to spectrogram-based classification [37,38,40,48,49,50].
- **Implement Cross-Validation and Overfitting Mitigation:** To ensure robust evaluation and improve model generalization, this study employs 5-fold cross-validation during training. This technique systematically partitions the dataset into five equal subsets (folds), where the model is trained on four folds and tested on the remaining one. The process is repeated five times, with each fold serving as the test set once. The final performance metrics are obtained by averaging the results across all runs. This approach mitigates overfitting and provides a more reliable estimate of the model’s performance on unseen data.
  In addition to cross-validation, transfer learning from pretrained ImageNet models was utilized to leverage well-generalized visual features, reducing the need for training from scratch. Furthermore, early stopping was applied during training to halt the process once the validation loss plateaued, and mini-batch training was used to stabilize updates and prevent overfitting in small batches. These combined strategies enhanced the robustness and generalization capability of the CNN architectures used in this study.
  To assess classification reliability, multiple evaluation metrics are employed:
  – **Accuracy**: Measures the proportion of correctly classified firearm sounds.
  – **Precision**: Evaluates the model’s ability to correctly identify specific firearm types, minimizing false positives.
  – **Recall (Sensitivity)**: Assesses the model’s effectiveness in detecting all instances of a given firearm sound, minimizing false negatives.
  – **F1-Score**: Provides a balanced evaluation by considering both precision and recall, making it particularly useful when dealing with imbalanced datasets.
  – **Confusion Matrix**: Analyzes misclassifications by providing insights into false positives and false negatives, helping identify areas for improvement in feature extraction or model training.
  By integrating **5-fold cross-validation** and comprehensive performance metrics, this study ensures that the trained models are robust, reliable, and capable of accurately classifying firearm sounds in semi-controlled outdoor environments.

## 4. Results and Discussion

This study introduces a robust framework for firearm sound classification by integrating advanced audio signal processing techniques with deep convolutional neural networks (CNNs). The proposed system comprises three main stages: audio preprocessing, spectrogram-based feature extraction, and deep-learning-driven classification. In particular, gunshot audio recordings are converted into time-frequency representations—spectrograms—which serve as input to pretrained CNN models. To enhance generalizability and minimize overfitting, k-fold cross-validation is employed throughout training and evaluation.

The classification task targets four firearm types: Smith & Wesson .38 caliber, Glock 17 Gen3 9 mm, Remington Model 870 12-gauge, and Ruger AR-556 .223 caliber. The dataset is derived from the work of Kabealo et al. [13], which features recordings captured in semi-controlled outdoor environments using multiple edge devices to reflect diverse acoustic conditions.

Transfer learning is applied using several established CNN architectures—GoogLeNet, Inception-v3, InceptionResNetV2, ResNet18, ResNet50, and ResNet101—originally trained on the ImageNet dataset. These models are fine-tuned for spectrogram classification by replacing their final layers with new fully connected, softmax, and classification layers, assigning a WeightLearnRateFactor and BiasLearnRateFactor of 20. Input images are resized to 224×224 for GoogLeNet and ResNet variants, and to 299×299 for Inception-based models. All models are trained using the Adam optimizer with a batch size of 32, an initial learning rate of 0.0001, and a maximum of 500 epochs.

In the following section, we present and analyze the results obtained from applying 12 distinct spectrogram types as input features. Each spectrogram is examined individually to assess its impact on classification accuracy across different CNN models. This comparative analysis provides valuable insights into the discriminative effectiveness of various time-frequency representations for firearm sound classification.

### 4.1. Performance Metrics for Bark Spectrogram with CNN Architectures

The experiment tested various CNN architectures—GoogLeNet, InceptionResNetV2, Inception-v3, ResNet18, ResNet50, and ResNet101—on a classification task using Bark spectrograms. The results show in Table 3 that all models struggled to achieve strong performance, with none exceeding 44% accuracy. ResNet18 delivered the best accuracy at 43.81%, suggesting limited capability to generalize from the data. GoogLeNet and InceptionResNetV2 showed perfect precision scores (1.0000), but their recall values were extremely low (0.0700 and 0.0500, respectively). This indicates that while the models rarely predicted false positives, they also failed to detect most actual positives. Their F1-scores were also very low, highlighting a lack of balance between precision and recall.

Inception-v3 performed slightly better than the previous two, with a more moderate precision (0.2819) and higher recall (0.4200), resulting in an F1-score of 0.3374. While still underwhelming, this balance shows marginal improvement in capturing true positives. ResNet18 stood out for its more balanced performance, with a precision of 0.4050 and recall of 0.4852, giving it the highest F1-score at 0.4414. It also had a solid specificity of 0.7825 and AUC of 0.6714, which suggests better overall class separation than other models. ResNet50 and ResNet101 had high specificity (above 0.92), but their recall and F1-scores were low, showing they were heavily biased toward negative predictions. These models would miss many true positives, which is problematic for most real-world classification tasks.

In summary, the Bark spectrogram alone does not appear to provide enough discriminative power for CNN architectures to perform reliably. Although ResNet18 showed relative promise, its accuracy and F1-score are still below acceptable thresholds for deployment. The dataset or feature type may be inadequate, or the task may require more sophisticated model tuning or feature engineering.

Based on these results, the use of Bark spectrograms with standard CNN architectures is not currently suitable for practical applications. Further improvements in data preprocessing, feature selection, or model design would be necessary before these systems could be considered viable.

### 4.2. Performance Metrics for Chroma Spectrogram with CNN Architectures

As shown in Table 4, the experiment evaluated the performance of various CNN architectures—GoogLeNet, InceptionResNetV2, Inception-v3, ResNet18, ResNet50, and ResNet101—using Chroma spectrograms as input features. Across the board, the models achieved strong performance, with all accuracies exceeding 84%, suggesting that Chroma spectrograms provide highly informative input representations for this classification task. InceptionResNetV2 achieved the highest accuracy at 88.92%, closely followed by ResNet50 at 88.78% and Inception-v3 at 88.32%. These results indicate that deeper and more complex architectures are particularly well-suited to learning from Chroma-based features.

In terms of precision, all models scored above 0.85, with InceptionResNetV2 leading at 0.9231. This high precision reflects a strong ability to minimize false positives. Recall values were similarly high, ranging from 0.7800 (GoogLeNet) to 0.8800 (Inception-v3 and ResNet50), showing that the models were also effective at detecting true positives. The F1-scores reflect balanced performance across precision and recall, with InceptionResNetV2 and ResNet50 both achieving 0.8800 or higher, making them strong candidates for deployment in scenarios requiring consistent classification reliability.

Specificity was also high, with all models above 0.95, indicating minimal false positive rates. The lowest FPR was recorded by InceptionResNetV2 at just 0.0213, showing excellent discriminatory power. The AUC scores further confirm these results, with all models scoring above 0.96. ResNet50 reached an AUC of 0.9796, while ResNet18 achieved a very close 0.9794 despite slightly lower accuracy. This suggests a strong ability to distinguish between classes under varying decision thresholds.

Based on the performance shown in Table 4, Chroma spectrograms used with CNN architectures can be confidently recommended for practical applications. Their performance is stable, accurate, and reliable, making them suitable for deployment in real-world systems that require effective and efficient audio-based classification.

### 4.3. Performance Metrics for Cochleagram Spectrogram with CNN Architectures

As shown in Table 5, the CNN architectures evaluated using Cochleagram spectrogram features achieved outstanding performance across all metrics. All models reached accuracy levels above 91%, with ResNet101 and ResNet50 performing the best at 93.9964% and 93.9516%, respectively. This indicates that Cochleagram features offer highly discriminative information suitable for CNN-based classification tasks.

Precision values across the models were consistently high, ranging from 0.9109 (ResNet18) to 0.9394 (InceptionResNetV2), indicating the models were very effective in minimizing false positives. Recall was also excellent across all architectures, with most models exceeding 0.91. ResNet50 achieved the highest recall at 0.9505, while ResNet101 was nearly identical at 0.9500. These high recall values suggest strong sensitivity to actual positive cases, a critical factor in many real-world applications.

F1-scores, which balance precision and recall, were equally impressive. All models scored above 0.91, with ResNet50 leading at 0.9412, followed by ResNet101 at 0.9314. These results confirm the ability of these networks to maintain balanced classification performance without favoring one metric at the expense of another.

Specificity was also strong, with all models scoring above 0.97. InceptionResNetV2 recorded the highest specificity at 0.9818, indicating its strong capability to correctly identify negative instances. The corresponding false positive rates (FPR) were extremely low, all under 0.03, reinforcing the overall reliability of the models in minimizing classification errors. The AUC scores, representing the overall ability of the models to distinguish between classes across all thresholds, were exceptionally high. Every model exceeded 0.98, with ResNet101 achieving the highest AUC of 0.9938, followed closely by ResNet50 at 0.9930. These near-perfect AUC values reflect consistent and robust performance.

Based on the metrics presented in Table 5, it is clear that Cochleagram spectrograms are highly effective features for CNN architectures. All evaluated models delivered high accuracy, balanced precision-recall performance, and excellent class separation. Therefore, Cochleagram-based CNN classification systems are fully suitable for real-world deployment and offer reliable and accurate performance across multiple evaluation criteria.

### 4.4. Performance Metrics for CQT Spectrogram with CNN Architectures

As shown in Table 6, the performance of CNN architectures using CQT (Constant-Q Transform) spectrogram features is remarkably high across all evaluated metrics. Every model achieved over 92% accuracy, with InceptionResNetV2 reaching the top accuracy of 95.8124%, followed closely by ResNet101 at 95.1647% and ResNet50 at 94.7835%. These results highlight the strong compatibility between CQT features and deep convolutional architectures.

Precision values are also notably high, ranging from 0.9029 (ResNet18) to 0.9691 (Inception-v3), showing that all models consistently predicted positive classes with minimal false positives. In terms of recall, performance was outstanding across the board, with ResNet50 and ResNet101 both achieving 0.9700. These models were particularly effective at identifying true positive instances, making them strong choices for tasks where missing positive cases would be costly.

F1-scores, which reflect the balance between precision and recall, further support the models’ robustness. Inception-v3 achieved the highest F1-score at 0.9543, followed closely by ResNet50 at 0.9463 and InceptionResNetV2 at 0.9447. These values confirm that the models did not trade off precision for recall, maintaining consistent strength in both areas.

Specificity was very high across all models, indicating strong performance in correctly classifying negative cases. Inception-v3 achieved the highest specificity at 0.9909, with a corresponding FPR of only 0.0092—the lowest among all models tested. These values suggest exceptional reliability in distinguishing between classes with minimal misclassification.

The AUC values underscore the overall excellence of these models. All AUC scores exceeded 0.989, with ResNet50 and ResNet101 both reaching the highest value of 0.9964. These near-perfect scores show that the models are highly effective across different classification thresholds, making them robust to varying decision criteria.

In conclusion, the results in Table 6 confirm that CQT spectrograms are highly effective input features for CNN-based classification. The consistently strong metrics across all architectures demonstrate reliable, accurate, and balanced performance. These models are well-suited for deployment in real-world applications where high precision and sensitivity are essential.

### 4.5. Performance Metrics for FFT Spectrogram with CNN Architectures

Table 7 presents the results of CNN models trained on FFT spectrogram features. The performance across all models is consistently strong, with accuracy ranging from 90.0728% (GoogLeNet) to 93.6021% (InceptionResNetV2). These values indicate that FFT spectrograms are effective for CNN-based classification, although they slightly trail the performance seen with CQT or Cochleagram features.

Precision values are generally high across all architectures, with InceptionResNetV2 achieving the best score at 0.9515, reflecting a strong ability to minimize false positives. Other models, such as GoogLeNet and ResNet50, also perform well in this regard, both exceeding 0.93. Recall scores, however, are slightly more varied. While GoogLeNet reached 0.8566 and ResNet50 achieved a solid 0.8784, InceptionResNetV2 dropped to 0.8290. This indicates that some models, despite high precision, may miss a notable number of true positive cases.

F1-scores, which provide a balanced measure of model performance, ranged from 0.8538 (ResNet18) to 0.9038 (ResNet50). ResNet50 stands out as offering the best trade-off between precision and recall, suggesting it is the most well-rounded performer among the group. Overall, most models delivered F1-scores above 0.87, demonstrating reliable classification performance.

Specificity was also robust across the board, with all models exceeding 0.96. InceptionResNetV2 had the highest specificity at 0.9701, accompanied by a low false positive rate of 0.0299. These metrics indicate that the models were generally effective at correctly identifying negative cases and limiting misclassifications.

AUC values for all architectures remained high, ranging from 0.9736 (GoogLeNet) to 0.9846 (InceptionResNetV2), further confirming strong overall classification ability. ResNet50 and Inception-v3 also performed well in this area, each scoring above 0.983, suggesting consistent discrimination across decision thresholds.

Considering the data in Table 7, CNNs trained with FFT spectrogram features deliver solid and reliable performance. Although marginally behind other spectrogram types in some metrics, FFT remains a viable and effective input representation for deep-learning-based audio classification tasks.

### 4.6. Performance Metrics for Mel Spectrogram with CNN Architectures

Performance results for CNN models using Mel spectrogram features are detailed in Table 8. The metrics indicate that Mel spectrograms are highly effective for audio classification tasks. All models surpassed 89% accuracy, with the best-performing architecture, ResNet50, achieving 94.8352%, followed closely by InceptionResNetV2 at 94.5173%. These figures suggest strong overall model generalization using Mel-based input.

Precision was consistently high across all models. InceptionResNetV2 and ResNet101 led with scores of 0.9500 and 0.9681, respectively, while the other models also maintained values above 0.90. These results show that false positive rates were kept low. Recall values were similarly strong, with ResNet50 achieving the highest at 0.9700, indicating a high sensitivity to true positive instances. Most models maintained recall above 0.90, reflecting dependable detection performance.

The F1-scores reinforce the strength of the models, balancing both precision and recall. ResNet50 again stands out with an F1-score of 0.9463, and InceptionResNetV2 is close behind at 0.9500. Even the lowest F1-score, from GoogLeNet, remains strong at 0.9026, confirming solid predictive consistency across the architectures.

Specificity values were high across all models, with ResNet101 achieving a peak score of 0.9909, indicating a very low false positive rate (FPR = 0.0091). Other models also maintained strong specificity, all above 0.96. This indicates reliability in correctly rejecting negative cases.

AUC scores offer further confirmation of overall performance, all exceeding 0.98. ResNet50 recorded the highest AUC at 0.9965, suggesting excellent class separation. InceptionResNetV2 and ResNet101 followed closely with AUCs of 0.9940 and 0.9945, respectively, reinforcing their effectiveness across decision thresholds.

Based on the results in Table 8, it is clear that CNN architectures paired with Mel spectrograms are highly effective for classification tasks. The consistent performance across accuracy, precision, recall, F1-score, specificity, and AUC makes this approach well-suited for practical deployment in real-world audio analysis systems.

### 4.7. Performance Metrics for MFCC Spectrogram with CNN Architectures

The performance metrics in Table 9 highlight the effectiveness of CNN architectures using MFCC (Mel-Frequency Cepstral Coefficient) spectrograms. All models achieved high classification accuracy, with InceptionResNetV2 leading at 94.7438%, followed closely by ResNet101 at 94.4135% and ResNet50 at 92.9236%. These results demonstrate that MFCCs are a strong input representation for deep-learning models in audio classification tasks.

Precision scores were consistently high across all architectures. InceptionResNetV2 reached the highest at 0.9515, while most others, such as ResNet101 (0.9412) and Inception-v3 (0.9381), also performed well. These values suggest the models were effective at minimizing false positives. Recall was equally strong, with values above 0.90 for every model. ResNet50 achieved the highest recall at 0.9604, followed closely by InceptionResNetV2 (0.9703), highlighting the models’ ability to detect true positives reliably.

F1-scores, which balance precision and recall, were uniformly strong. InceptionResNetV2 again led with 0.9608, confirming its well-rounded performance. ResNet101 (0.9458) and ResNet50 (0.9238) also demonstrated strong overall classification consistency. Even the lowest F1-score, from GoogLeNet at 0.9091, remained robust, indicating that all models maintained a reliable precision-recall balance.

Specificity scores were high, with most models scoring above 0.96. InceptionResNetV2 achieved 0.9848, and Inception-v3 followed with 0.9818, indicating strong performance in correctly identifying negative cases. Corresponding false positive rates (FPR) remained low, under 0.04 for all models, further demonstrating their classification stability.

AUC values reinforced these findings, with all architectures scoring above 0.988. InceptionResNetV2 achieved the highest AUC at 0.9973, showing near-perfect class separability. ResNet101 (0.9953) and ResNet50 (0.9935) were also excellent in this regard.

Overall, the results in Table 9 confirm that MFCC spectrograms are highly effective for CNN-based classification. The combination of high accuracy, balanced precision and recall, low false positive rates, and strong AUC scores makes this approach reliable and well-suited for deployment in real-world audio recognition systems.

### 4.8. Performance Metrics for Reassigned Spectrogram with CNN Architectures

Table 10 displays the results of CNN models trained on Reassigned spectrogram features, showing high performance across all evaluated metrics. All architectures achieved over 89% accuracy, with ResNet101 performing best at 93.6214%, followed by InceptionResNetV2 at 93.1639%. These results suggest that the Reassigned spectrogram is an effective input representation for CNN-based classification.

Precision values were strong across the board, ranging from 0.8611 (ResNet18) to 0.9406 (ResNet101). The higher scores for InceptionResNetV2, Inception-v3, and ResNet101 indicate that these models effectively minimized false positives. Recall values were even more consistent, with all models exceeding 0.92. ResNet18, despite a lower precision, reached a recall of 0.9300, highlighting its ability to identify true positives effectively.

F1-scores, which measure the balance between precision and recall, were all above 0.89. ResNet101 achieved the highest F1-score at 0.9406, with InceptionResNetV2 and Inception-v3 following closely at 0.9353 and 0.9307, respectively. Even GoogLeNet and ResNet50 maintained respectable F1-scores above 0.90, showing reliable overall classification performance.

Specificity scores were similarly high, with all models above 0.95. ResNet101 again led with 0.9817, closely followed by InceptionResNetV2 at 0.9787. These values reflect strong performance in correctly identifying negative instances. Corresponding false positive rates were all low, ranging from 0.0183 to 0.0455, with most models staying under 0.03.

AUC values further confirmed the models’ strength, with all architectures achieving scores above 0.985. ResNet101 reached the highest AUC at 0.9956, with Inception-v3 and ResNet50 also performing exceptionally well. These results suggest excellent class separation and model robustness across decision thresholds.

In summary, the performance metrics in Table 10 demonstrate that Reassigned spectrograms are highly suitable for CNN-based classification. The models consistently achieved strong accuracy, balanced precision-recall performance, and high specificity and AUC values. These results indicate that reassigned spectrograms offer a reliable and effective approach for real-world audio classification tasks.

### 4.9. Performance Metrics for Spectral Contrast Spectrogram with CNN Architectures

Table 11 presents the performance of CNN architectures trained on Spectral Contrast spectrogram features for firearm sound classification. Among the models evaluated, ResNet101 achieved the highest accuracy at 74.8619%, followed closely by InceptionResNetV2 (74.6378%) and ResNet18 (72.3024%). These results suggest that architectures incorporating residual connections, and especially those combining residual and inception modules, are more effective at extracting relevant patterns from spectral contrast inputs.

Despite these relatively strong results for a contrast-based representation, the overall accuracy of all models remains lower than what was observed with other spectrogram types, such as Mel or MFCC. Precision scores ranged from 0.6941 (GoogLeNet) to 0.7742 (ResNet18), while recall values ranged from 0.5842 (GoogLeNet) to 0.7426 (ResNet101). This disparity highlights the difficulty CNNs face in capturing the necessary temporal and spectral information from spectral contrast features alone.

F1-scores reflect similar trends. ResNet18 achieved the highest F1-score at 0.7461, followed closely by InceptionResNetV2 (0.7423) and ResNet101 (0.7389). These values suggest that while the models are reasonably balanced in their precision and recall, their overall performance is still constrained by the limitations of the input representation. The results also indicate that deeper models do not consistently outperform shallower ones, pointing to the importance of architectural design over sheer depth when working with contrast-based inputs.

Specificity scores remained high across the board, with values ranging from 0.9119 to 0.9360, indicating that the models were generally good at identifying negative cases. However, false positive rates (FPR) were noticeably higher than those seen with other spectrogram types, reaching up to 0.0882 in the case of ResNet50. This contributes to lower reliability and robustness in classification.

AUC values ranged from 0.8685 (GoogLeNet) to 0.9268 (ResNet101), demonstrating moderate class separability. Although these scores confirm that CNNs trained on spectral contrast features have some discriminative power, they are still inferior to models trained on richer time-frequency representations.

In summary, while Spectral Contrast spectrograms can provide moderate performance for firearm sound classification using CNNs, they do not offer the same robustness or reliability as other spectrogram types. These findings suggest that future approaches should consider combining spectral contrast with other features or adopting alternative architectures to improve classification outcomes.

### 4.10. Performance Metrics for STFT Spectrogram with CNN Architectures

Table 12 summarizes the performance of CNN architectures trained on STFT (Short-Time Fourier Transform) spectrograms. The results demonstrate that STFT is a highly effective input representation for firearm sound classification using deep learning. All models achieved high accuracy, with InceptionResNetV2 leading at 94.7901%, followed by ResNet101 (93.1165%) and Inception-v3 (93.5834%). These results highlight the effectiveness of both residual and inception-based architectures in modeling time-frequency representations.

Precision scores were strong across all models, with Inception-v3 achieving the highest at 0.9681. InceptionResNetV2 and ResNet50 also performed well, with precision values above 0.92. Recall scores were equally impressive. InceptionResNetV2 achieved the highest recall at 0.9800, indicating an exceptional ability to detect true positive instances. GoogLeNet, despite being a shallower architecture, also showed strong recall at 0.9400 but suffered from lower precision (0.7402), leading to a more modest F1-score.

F1-scores for most models were high, reflecting a strong balance between precision and recall. InceptionResNetV2 stood out with an F1-score of 0.9561, followed closely by ResNet50 (0.9412) and Inception-v3 (0.9381). These results affirm that deeper models with sophisticated architectures are capable of extracting rich and meaningful patterns from STFT spectrograms.

Specificity values were uniformly high, with Inception-v3 achieving the best score at 0.9909. Most other models also exceeded 0.96, showing that false positive rates were kept low. GoogLeNet was the exception, with a specificity of 0.8994 and a relatively high FPR of 0.1006, suggesting a higher rate of misclassifications compared to the deeper models.

AUC scores support the overall conclusion of strong model performance. InceptionResNetV2 achieved the highest AUC at 0.9976, indicating near-perfect class discrimination. All other models recorded AUC values above 0.97, reinforcing their ability to maintain high performance across different classification thresholds.

In conclusion, the data in Table 12 confirms that STFT spectrograms, when paired with advanced CNN architectures, provide an excellent foundation for accurate and reliable firearm sound classification. These models consistently deliver high precision, recall, F1-score, and AUC, making them well-suited for real-world implementation in intelligent acoustic recognition systems.

### 4.11. Performance Metrics for Wavelet Spectrogram with CNN Architectures

Table 13 presents the evaluation results of CNN architectures trained on Wavelet spectrogram features for firearm sound classification. The overall performance is strong across all models, with accuracy values ranging from 84.5927% (ResNet18) to 92.8849% (ResNet101). These results confirm that Wavelet-based time-frequency representations are effective for deep-learning-based audio classification, offering a good balance between time and frequency localization.

Precision scores across the architectures were consistently high, with ResNet101 achieving the best value at 0.9406. Other models, including ResNet50 (0.9126), Inception-v3 (0.9167), and GoogLeNet (0.9011), also showed strong performance in minimizing false positives. Recall scores were similarly robust, with all models exceeding 0.82. InceptionResNetV2, ResNet50, and ResNet101 all reached a recall of 0.9400 or above, indicating excellent sensitivity to true positive instances.

F1-scores reflect the models’ balanced performance. ResNet101 stood out with a perfect balance of 0.9406 in both precision and recall, leading to a strong F1-score. ResNet50 and InceptionResNetV2 followed closely with F1-scores of 0.9261 and 0.9216, respectively. Even the lowest score, from GoogLeNet at 0.8586, was still quite competitive, reaffirming the effectiveness of Wavelet spectrograms as input features.

Specificity values were high for all models, indicating a strong ability to correctly identify negative cases. ResNet101 achieved the highest specificity at 0.9817, while most other models were also above 0.96. The corresponding false positive rates remained low, ranging from 0.0183 (ResNet101) to 0.0424 (ResNet18), supporting the models’ reliability in avoiding misclassifications.

AUC scores further validate the models’ classification capabilities. All models scored above 0.977, with ResNet101 reaching the highest AUC at 0.9933. This indicates excellent class separability and robustness across decision thresholds. ResNet50 and InceptionResNetV2 also performed exceptionally well, with AUC values above 0.99.

In summary, the results in Table 13 demonstrate that Wavelet spectrograms, when paired with modern CNN architectures, offer highly reliable and accurate performance for firearm sound classification. These models show a strong balance of precision, recall, and specificity, making Wavelet-based CNN classifiers suitable for real-world applications in intelligent acoustic detection systems.

### 4.12. Discussion on CNN Performance Variation by Spectrogram Type

**Figure 2:**
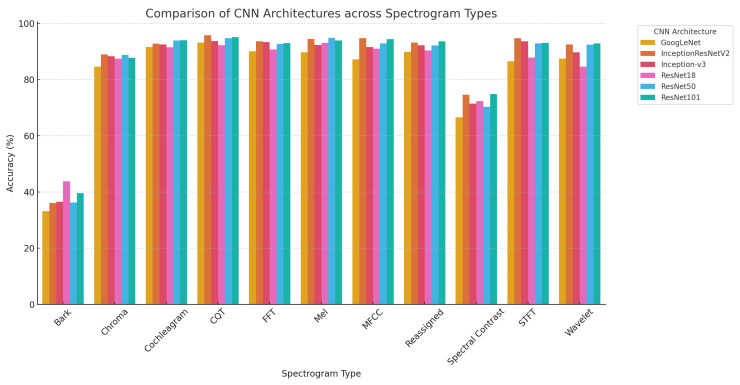
Comparison of CNN Architectures across Spectrogram Types.

This study presents a rigorous comparative analysis of convolutional neural network (CNN) architectures applied to a comprehensive set of time-frequency spectrogram representations for firearm sound classification. The experimental findings, derived from performance metrics detailed in Table 6, Table 7, Table 8, Table 9, Table 10, Table 11, Table 12 and Table 13 and visualized in Figure 2, offer critical insights into the interplay between spectrogram encoding techniques and deep-learning model performance.

The results affirm that image-based spectrogram representations are highly effective for firearm audio recognition, validating their potential for deployment in intelligent acoustic surveillance systems aimed at mitigating firearm-related incidents. Among the evaluated features, the Constant-Q Transform (CQT) demonstrated superior performance, particularly when integrated with advanced hybrid architectures such as InceptionResNetV2 and ResNet101. These models achieved classification accuracies of 95.8124% and 95.1647%, respectively, underscoring the efficacy of combining logarithmic frequency scaling with deep residual-inception frameworks for capturing complex spectral patterns.

Biologically inspired representations such as Cochleagram also yielded high classification performance, with ResNet101 achieving an accuracy of 93.9964%. Furthermore, MFCC and Mel spectrograms—widely used in speech and environmental sound recognition—maintained consistently strong results, with multiple models exceeding 94% accuracy. These findings reaffirm their suitability for firearm detection tasks.

Conversely, Bark and Spectral Contrast spectrograms exhibited markedly lower performance. Bark, despite its psychoacoustic basis, reached a maximum accuracy of only 43.8075% (ResNet18), suggesting limited utility without further enhancement. Spectral Contrast, while moderately effective, lacked the feature richness required to support robust classification across CNN variants.

The comparative evaluation of CNN architectures revealed that deeper and hybrid models, notably ResNet50, ResNet101, and InceptionResNetV2, outperformed shallower networks like GoogLeNet. The consistent superiority of these models, reflected in their higher accuracy, recall, and AUC values, highlights the importance of architectural depth and modular design for modeling complex audio representations. Inception-based models also performed favorably with high-detail spectrograms such as Reassigned and Spectral Contrast, further confirming their flexibility.

These findings are fully consistent with the stated Research Objectives and Scope of Research, successfully demonstrating the effectiveness of CNN-based classification across diverse spectrogram inputs. The study achieves its goals by identifying optimal feature-model combinations and contributes a valuable foundation for the development of practical, deployable firearm detection systems.

While our study provides a thorough comparative analysis across different spectrogram types and CNN architectures, it does not currently employ explicit feature fusion strategies within the network architectures. Recent advances in object detection and scene understanding have demonstrated that multi-scale feature fusion can significantly improve performance, especially when input representations vary in scale or frequency emphasis.

Notable approaches such as Feature Pyramid Networks (FPN) [51], Zoom Text Detector [52], Asymptotic Feature Pyramid Networks (AFPN) [53], and CM-Net [54] showcase effective mechanisms for aggregating hierarchical feature maps, enhancing the model’s ability to capture fine-grained and contextual patterns.

In the context of firearm sound classification, integrating multi-spectrogram fusion—e.g., combining CQT and Mel features—may allow CNNs to extract both perceptual and pitch-related features simultaneously. This could be implemented via channel-wise concatenation, attention modules, or late-fusion ensemble strategies. Future work could investigate embedding such fusion mechanisms into the CNN backbone to further boost classification robustness, especially under real-world noisy conditions or ambiguous gunshot signatures.

## 5. Conclusions

This study proposed a deep-learning framework for firearm sound classification based on image-based time-frequency feature extraction. By transforming raw gunshot audio recordings into 12 types of spectrograms—converted into RGB images—the system enables the application of powerful computer vision techniques to acoustic data. These spectrograms serve as two-dimensional feature maps, allowing pretrained convolutional neural networks (CNNs), originally designed for image recognition, to effectively learn firearm-specific acoustic patterns.

Among the spectrogram types evaluated, perceptually motivated representations such as Constant-Q Transform (CQT), Mel, and Cochleagram consistently delivered superior classification performance. When combined with deep and hybrid CNN architectures—particularly ResNet101 and InceptionResNetV2—these spectrograms achieved accuracies above 95%, F1-scores exceeding 0.94, and AUC values approaching 0.996. These results highlight the importance of both feature representation and model architecture in building robust and accurate gunshot classification systems.

Importantly, this work demonstrates that transforming audio signals into visual representations is not merely a preprocessing step but a powerful strategy for feature extraction. This paradigm effectively bridges the domains of audio signal processing and computer vision, supporting the development of intelligent acoustic surveillance and forensic analysis tools.

**Future Work.** Moving forward, future research will focus on enhancing real-time classification by integrating the proposed pipeline into edge computing platforms, enabling low-latency alerts in smart surveillance systems. We also aim to explore sequential modeling approaches to capture temporal patterns in bursts of gunfire and to distinguish gunshots from acoustically similar events such as fireworks or vehicle backfires. Expanding the dataset to include more firearm types, varied recording conditions, and background noise will further improve model generalization.

In addition, several methodological improvements will be prioritized in response to limitations identified in this study. First, we plan to conduct an ablation study to validate the impact of different colormap choices by comparing RGB (Jet/Viridis), grayscale, and multi-channel spectrogram encodings. Second, we aim to perform a sensitivity analysis on spectrogram parameters—such as hop length, window size, FFT size, and Mel filterbank configuration—to assess their influence on model performance and reproducibility. Third, future work will benchmark the proposed CNN-based pipeline against alternative learning paradigms, including raw waveform models (e.g., 1D CNNs), sequential models (e.g., LSTM/GRU), and attention-based models such as Audio Spectrogram Transformers (AST), Perceiver AR, and HTS-AT. We also intend to evaluate audio-specific architectures like LEAF and HEARNet to assess their suitability for firearm classification tasks.

Furthermore, we plan to investigate lightweight CNN and transformer-based architectures, as well as self-supervised learning methods, to support deployment in resource-constrained environments. Although high-performing models such as ResNet101 are effective, their higher inference time and computational demand highlight the need for lightweight, optimized models for real-time surveillance applications. Finally, to promote scientific transparency, we intend to apply statistical tests (e.g., ANOVA, t-tests) and publicly release the spectrogram generation pipeline and training code. While our results are promising, future studies should also explore how these models perform in uncontrolled, noise-heavy urban environments to ensure practical applicability in real-world scenarios.

## Figures and Tables

**Table 1:** Spectrogram representations of gunshot sounds from different firearm types.

Type of Spectrogram	Smith & Wesson (.38)	Glock 17 Gen3 (9 mm)	Remington 870 (12-Gauge)	Ruger AR-556 (.223)
Bark	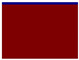	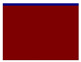	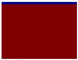	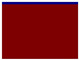
Chroma	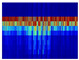	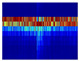	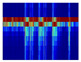	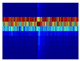
Cochleagram	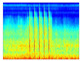	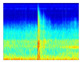	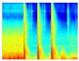	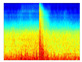
CQT	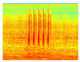	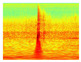	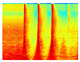	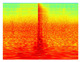
FFT	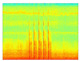	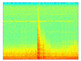	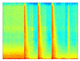	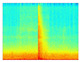
Mel	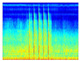	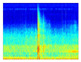	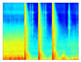	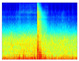
Reassigned	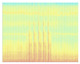	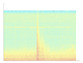	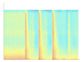	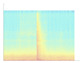
Spectral Contrast	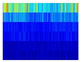	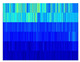	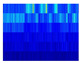	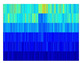
STFT	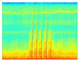	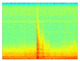	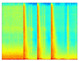	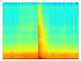
Wavelet	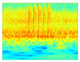	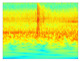	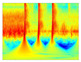	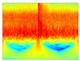

**Table 2:** Distribution of gunshot audio files by firearm type in the dataset.

Gun Type	Number of Files
Glock 17 Gen3 (9 mm)	730
Remington Model 870 12-gauge	466
Ruger AR-556 .223 caliber	728
Smith & Wesson .38 caliber	519

**Table 3:** Performance metrics for Bark spectrogram with CNN architectures.

CNN Architecture	Accuracy (%)	Precision	Recall	F1-Score	Specificity	FPR	AUC
GoogLeNet	33.1947	1.0000	0.0700	0.1308	1.0000	0.0000	0.5457
InceptionResNetV2	36.0389	1.0000	0.0500	0.0952	1.0000	0.0000	0.6251
Inception-v3	36.5032	0.2819	0.4200	0.3374	0.6738	0.3262	0.5919
ResNet18	43.8075	0.4050	0.4852	0.4414	0.7825	0.2175	0.6714
ResNet50	36.2241	0.4681	0.2178	0.2973	0.9238	0.0762	0.6703
ResNet101	39.5793	0.5926	0.1584	0.2500	0.9666	0.0334	0.6201

**Table 4:** Performance metrics for Chroma spectrogram with CNN architectures.

CNN Architecture	Accuracy (%)	Precision	Recall	F1-Score	Specificity	FPR	AUC
GoogLeNet	84.5882	0.8571	0.7800	0.8168	0.9607	0.0393	0.9654
InceptionResNetV2	88.9205	0.9231	0.8400	0.8796	0.9787	0.0213	0.9800
Inception-v3	88.3167	0.8889	0.8800	0.8844	0.9666	0.0334	0.9776
ResNet18	87.4308	0.8614	0.8614	0.8614	0.9573	0.0427	0.9794
ResNet50	88.7813	0.8800	0.8800	0.8800	0.9635	0.0365	0.9796
ResNet101	87.7104	0.8936	0.8317	0.8615	0.9697	0.0303	0.9734

**Table 5:** Performance metrics for Cochleagram spectrogram with CNN architectures.

CNN Architecture	Accuracy (%)	Precision	Recall	F1-Score	Specificity	FPR	AUC
GoogLeNet	91.5342	0.9126	0.9400	0.9261	0.9726	0.0274	0.9895
InceptionResNetV2	92.8305	0.9394	0.9208	0.9300	0.9818	0.0182	0.9907
Inception-v3	92.5038	0.9200	0.9109	0.9154	0.9757	0.0243	0.9899
ResNet18	91.4431	0.9109	0.9109	0.9109	0.9726	0.0274	0.9886
ResNet50	93.9516	0.9320	0.9505	0.9412	0.9787	0.0213	0.9930
ResNet101	93.9964	0.9135	0.9500	0.9314	0.9726	0.0274	0.9938

**Table 6:** Performance metrics for CQT spectrogram with CNN architectures.

CNN Architecture	Accuracy (%)	Precision	Recall	F1-Score	Specificity	FPR	AUC
GoogLeNet	93.2031	0.9307	0.9307	0.9307	0.9787	0.0213	0.9926
InceptionResNetV2	95.8124	0.9592	0.9307	0.9447	0.9880	0.0121	0.9957
Inception-v3	93.7149	0.9691	0.9400	0.9543	0.9909	0.0092	0.9941
ResNet18	92.1816	0.9029	0.9208	0.9118	0.9698	0.0302	0.9894
ResNet50	94.7835	0.9238	0.9700	0.9463	0.9756	0.0244	0.9964
ResNet101	95.1647	0.9151	0.9700	0.9418	0.9726	0.0274	0.9964

**Table 7:** Performance metrics for FFT spectrogram with CNN architectures.

CNN Architecture	Accuracy (%)	Precision	Recall	F1-Score	Specificity	FPR	AUC
GoogLeNet	90.0728	0.9303	0.8566	0.8913	0.9625	0.0375	0.9736
InceptionResNetV2	93.6021	0.9515	0.8290	0.8850	0.9701	0.0299	0.9846
Inception-v3	93.3617	0.9316	0.8318	0.8785	0.9669	0.0331	0.9831
ResNet18	90.7324	0.9012	0.8110	0.8538	0.9607	0.0393	0.9815
ResNet50	92.7176	0.9307	0.8784	0.9038	0.9674	0.0326	0.9836
ResNet101	93.0083	0.9254	0.8670	0.8951	0.9651	0.0349	0.9815

**Table 8:** Performance metrics for Mel spectrogram with CNN architectures.

CNN Architecture	Accuracy (%)	Precision	Recall	F1-Score	Specificity	FPR	AUC
GoogLeNet	89.6725	0.9263	0.8800	0.9026	0.9788	0.0212	0.9853
InceptionResNetV2	94.5173	0.9500	0.9500	0.9500	0.9849	0.0152	0.9940
Inception-v3	92.3218	0.9039	0.9307	0.9171	0.9695	0.0305	0.9893
ResNet18	93.0614	0.9208	0.9208	0.9208	0.9758	0.0242	0.9899
ResNet50	94.8352	0.9238	0.9700	0.9463	0.9756	0.0244	0.9965
ResNet101	93.9578	0.9681	0.9010	0.9333	0.9909	0.0091	0.9945

**Table 9:** Performance metrics for MFCC spectrogram with CNN architectures.

CNN Architecture	Accuracy (%)	Precision	Recall	F1-Score	Specificity	FPR	AUC
GoogLeNet	87.2064	0.9184	0.9000	0.9091	0.9757	0.0243	0.9886
InceptionResNetV2	94.7438	0.9515	0.9703	0.9608	0.9848	0.0152	0.9973
Inception-v3	91.5732	0.9381	0.9010	0.9192	0.9818	0.0182	0.9915
ResNet18	90.9724	0.9200	0.9200	0.9200	0.9756	0.0244	0.9900
ResNet50	92.9236	0.8899	0.9604	0.9238	0.9635	0.0365	0.9935
ResNet101	94.4135	0.9412	0.9505	0.9458	0.9817	0.0183	0.9953

**Table 10:** Performance metrics for Reassigned spectrogram with CNN architectures.

CNN Architecture	Accuracy (%)	Precision	Recall	F1-Score	Specificity	FPR	AUC
GoogLeNet	89.9023	0.8857	0.9208	0.9029	0.9635	0.0365	0.9884
InceptionResNetV2	93.1639	0.9307	0.9400	0.9353	0.9787	0.0213	0.9927
Inception-v3	92.2735	0.9216	0.9400	0.9307	0.9756	0.0244	0.9930
ResNet18	90.3246	0.8611	0.9300	0.8942	0.9546	0.0455	0.9856
ResNet50	92.1387	0.9118	0.9300	0.9208	0.9726	0.0274	0.9945
ResNet101	93.6214	0.9406	0.9406	0.9406	0.9817	0.0183	0.9956

**Table 11:** Performance metrics for Spectral contrast spectrogram with CNN architectures.

CNN Architecture	Accuracy (%)	Precision	Recall	F1-Score	Specificity	FPR	AUC
GoogLeNet	66.5732	0.6941	0.5842	0.6344	0.9210	0.0790	0.8685
InceptionResNetV2	74.6378	0.7660	0.7200	0.7423	0.9331	0.0669	0.9173
Inception-v3	71.3749	0.7391	0.6733	0.7047	0.9268	0.0732	0.9049
ResNet18	72.3024	0.7742	0.7200	0.7461	0.9360	0.0640	0.9144
ResNet50	70.3047	0.7129	0.7129	0.7129	0.9119	0.0882	0.9086
ResNet101	74.8619	0.7353	0.7426	0.7389	0.9179	0.0821	0.9268

**Table 12:** Performance metrics for STFT spectrogram with CNN architectures.

CNN Architecture	Accuracy (%)	Precision	Recall	F1-Score	Specificity	FPR	AUC
GoogLeNet	86.5432	0.7402	0.9400	0.8282	0.8994	0.1006	0.9709
InceptionResNetV2	94.7901	0.9333	0.9800	0.9561	0.9787	0.0213	0.9976
Inception-v3	93.5834	0.9681	0.9100	0.9381	0.9909	0.0091	0.9936
ResNet18	87.8506	0.8900	0.8812	0.8856	0.9668	0.0332	0.9839
ResNet50	92.9239	0.9231	0.9600	0.9412	0.9757	0.0243	0.9949
ResNet101	93.1165	0.9065	0.9604	0.9327	0.9698	0.0302	0.9939

**Table 13:** Performance metrics for Wavelet spectrogram with CNN architectures.

CNN Architecture	Accuracy (%)	Precision	Recall	F1-Score	Specificity	FPR	AUC
GoogLeNet	87.4312	0.9011	0.8200	0.8586	0.9727	0.0273	0.9777
InceptionResNetV2	92.5543	0.9039	0.9400	0.9216	0.9697	0.0303	0.9929
Inception-v3	89.6635	0.9167	0.8628	0.8889	0.9758	0.0242	0.9838
ResNet18	84.5927	0.8614	0.8614	0.8614	0.9576	0.0424	0.9794
ResNet50	92.4096	0.9126	0.9400	0.9261	0.9727	0.0273	0.9910
ResNet101	92.8849	0.9406	0.9406	0.9406	0.9817	0.0183	0.9933

## Data Availability

The dataset used in this study is publicly available in the Data in Brief journal under the title “A multi-firearm, multi-orientation audio dataset of gunshots“ by Ruksana Kabealo et al. [13], 2023. It can be accessed at https://doi.org/10.1016/j.dib.2023.109091.
